# Effects of the standardized ileal digestible lysine to metabolizable energy ratio on performance and carcass characteristics of growing-finishing pigs

**DOI:** 10.1186/2049-1891-3-9

**Published:** 2012-03-01

**Authors:** Pengfei Li, Zhikai Zeng, Ding Wang, Lingfeng Xue, Rongfei Zhang, Xiangshu Piao

**Affiliations:** 1 Ministry of Agriculture Feed Industry Centre, State Key Laboratory of Animal Nutrition, China Agricultural University, Beijing 100193, China

**Keywords:** carcass characteristics, performance, growing-finishing pigs, metabolizable energy, standardized ileal digestible lysine

## Abstract

A total of 2,121 growing-finishing pigs (Duroc × Landrace × Large White) were utilized in six experiments conducted to determine the effects of different ratios of standardized ileal digestible lysine (SID-Lys) to metabolizable energy (ME) on the performance and carcass characteristics of growing-finishing pigs. Exps. 1 (30 to 50 kg), 2 (52 to 70 kg) and 3 (81 to 104 kg) were conducted to find an optimum ME level and then this level was used in Exps. 4 (29 to 47 kg), 5 (54 to 76 kg) and 6 (84 to 109 kg) to test the response of pigs to different ratios of SID-Lys:ME. In Exps.1 to 3, four treatments were used consisting of diets with a formulated ME content of 3.1, 3.2, 3.3 or 3.4 in Exps. 1 and 2 while Exp. 3 used 3.05, 3.15, 3.25 or 3.35 Mcal/kg. A constant SID-Lys:ME ratio of 2.6, 2.3 or 2.0 g/Mcal was used in Exps. 1, 2 and 3, respectively. Weight gain was significantly increased with increasing energy level in Exp.1 while weight gain was unaltered in Exps. 2 and 3. For all three experiments, feed intake was decreased (*P *< 0.05) and feed efficiency was improved (*P *< 0.05) with increasing energy level. Tenth rib back fat thickness linearly increased (*P *< 0.05) with increasing energy level. In Exps. 4 to 6, five treatments were used consisting of diets with a SID-Lys:ME ratio of 2.4, 2.6, 2.8, 3.0 or 3.2 in Exp. 1, 2.1, 2.3, 2.5, 2.7, 2.9 or 3.2 in Exp. 2 and 1.8, 2.0, 2.2, 2.4, or 2.6 in Exp. 3. A constant ME level 3.2, 3.2 and 3.05 Mcal/kg was used in Exps. 1, 2 and 3, respectively (selected based on the results of weight gain). For all three experiments, weight gain increased (*P *< 0.05) and feed efficiency improved linearly (*P *< 0.05) as the SID-Lys:ME ratio increased. Tenth rib back fat thickness linearly decreased (*P *< 0.05) as the SID-Lys:ME ratio increased. Based on a straight broken-line model, the estimated SID-Lys:ME ratio to maximize weight gain was 3.0, 2.43 and 2.2 for 29 to 47, 54 to76 and 84 to 109 kg of pigs, respectively.

## Background

Two important objectives in pig production are to maximum growth rate and improve the efficiency of nutrient utilization [1]. Dietary lysine is a key factor which influences the achievement of these objectives because it is the first limiting amino acid in diets fed to swine [2]. Dietary energy influences feed intake in growing-finishing pigs fed *ad libitum *[3,4], so that a pig's amino acid intake may be altered as the energy content of the diet changes. Therefore, it is necessary to maintain an optimum lysine to energy ratio when the amino acid or energy content of the diet increases [1,3].

Previous studies have been conducted using variable ratios of lysine to energy [1]. Cho et al. [5] observed that the best lysine to digestible energy (DE) ratio for maximum amino acid digestibility of the pig is 2.4 g total lysine/Mcal DE for barrows (Landrace × Yorshire × Duroc; 64 kg). Chang et al. [6] showed that the optimal lysine to DE ratios were 3.2 and 3.8 g total lysine/Mcal DE for barrows and gilts (Landrace × Yorshire × Duroc; 16 to 57 kg), respectively. Bikker et al. [7] suggested that 2.5 g apparent ileal lysine/Mcal DE was required to optimize performance for lean gilts (20 to 45 kg). Factors which are responsible for the variation include environment and advance in the genetics of modern pigs [8,9]. Previous recommendation [2] is no longer fulfilling the desperate need of animal industry and production in China. Therefore, it is necessary for us to further research the lysine and energy requirement of growing-finishing pigs (Duroc × Landrace × Large White) under commercial pig farms condition in Hebei province, thereby contributing to the solid construction of Chinese feeding criterion.

Ileal digestibility coefficients for amino acids can be expressed as apparent, standardized or true. However, apparent ileal digestibility values are not always additive in a mixed diet [10]. In addition, it is difficult to measure specific endogenous amino acid losses and as a result, true ileal digestibility is not very practical for use in routine diet formulation [10]. Therefore, standardized ileal digestible values have been suggested as the best choice to be used for routine feed formulation [11] because it is additive in mixed diets and does not require the measurement of specific endogenous amino acid loss [12].

Besides the previous study on nursery pigs, the objective of this study was to determine the optimum ratio of SID-Lys:ME to maximize performance and carcass characteristics of growing-finishing pigs housed under commercial farm conditions.

## Materials and methods

### Animals and facilities

The experimental protocols and procedures used in these experiments were approved by the Institutional Animal Care and Use Committee of China Agricultural University (Beijing, China). The experiments were carried out at the Hebei Huailai Changfu Pig Culture Company (Hebei, China). All pigs (Duroc × Landrace × Large White) were housed in an all-in, all-out room in which the temperature was controlled between 17 and 22°C. The light schedule was 12 h light: 12 h dark. Pigs had free access to water and feed with diets provided in the form of a mash.

### Experimental diets and measurements

The ME content of the corn, soybean meal and wheat bran used in these experiments (Table 1) were determined previously in our laboratory (data not published). The ME content of soybean oil (calculated as 96% of DE) was obtained from Feeding Standard of Swine [2]. The SID lysine content of corn, soybean meal and wheat bran (Table 1) were determined in our laboratory using pigs surgically equipped with a T-cannula in the distal ileum (data not published). The basal ileal endogenous losses were measured by feeding a nitrogen-free diet. The SID lysine content of L-lysine HCL lysine was assumed to be 100%. The ME and SID lysine content of the experimental diets were calculated by multiplying the ME and SID content of the individual ingredients by their inclusion level in the diets and summing the product.

**Table 1:** ME and SID lysine values of individual ingredients used in experiments 1 to 6

	Corn	Soybean meal	Wheat bran	Soybean oil
Metabolizable energy^1^, Mcal/kg	3.39	3.59	2.18	8.40
Standardized ileal digestible lysine^2^, %	0.18	2.39	0.38	-

^1^The metabolizable energy content of corn, soybean meal and wheat bran were determined previously in our laboratory (data not published). Metabolizable energy of soybean oil (calculated as 96% of DE) was obtained from Feeding Standard of Swine [2].

^2^Standardized ileal digestible lysine was determined previously using pigs surgically equipped with a T-cannula in the distal ileum. The basal ileal endogenous losses were measured by feeding a nitrogen-free diet (data not published).

Six experiments were conducted to determine the effects of different ratios of standardized ileal digestible lysine (SID-Lys) to metabolizable energy (ME) on the performance and carcass characteristics of growing-finishing pigs during the growing, early finishing and late finishing periods. Exps. 1 (30 to 50 kg), 2 (52 to 70 kg) and 3 (81 to 104 kg) were conducted to find an optimum ME level and then this level was used in Exps. 4 (29 to 47 kg), 5 (54 to 76 kg) and 6 (84 to 109 kg) to test the response of pigs to different ratios of SID-Lys:ME.

For Exps. 1 to 3, a total of 360, 312 and 264 crossbred pigs (Duroc × Landrace × Large White), weighing 30.66 ± 4.55 kg, 51.98 ± 5.45 kg and 81.08 ± 8.40 kg of BW were assigned to one of four treatments. The four treatments consisted of diets with a formulated ME content of 3.1, 3.2, 3.3 or 3.4 in Exps. 1 and 2 while Exp. 3 used 3.05, 3.15, 3.25 or 3.35 Mcal/kg. A constant SID Lys:ME ratio of 2.6, 2.3 or 2.0 g/Mcal was used in Exps. 1, 2 and 3, respectively (Table 2). Other amino acids were balanced relative to lysine using crystalline amino acids in order to match the ideal amino acid profile of Feeding Standard of Swine [2].

**Table 2:** Ingredient and chemical composition of experimental diets used in experiments 1 to 3 (as-fed)

Item	ME level in 30 to 50 kg, Mcal/kg				ME level in 52 to 70 kg, Mcal/kg				ME level in 81 to 104 kg, Mcal/kg			
	
	3.10	3.20	3.30	3.40	3.10	3.20	3.30	3.40	3.05	3.15	3.25	3.35
Ingredient, %												
Corn	57.00	63.00	70.15	68.27	58.00	65.00	72.35	71.60	60.28	67.00	75.25	77.47
Soybean meal, (43% CP)	22.50	24.00	26.00	26.00	21.00	22.08	23.00	23.00	15.50	16.50	17.00	18.00
Wheat bran	16.97	9.36	0.00	0.00	17.75	9.50	1.00	0.00	21.05	13.31	4.28	0.00
Soybean oil	0.00	0.00	0.00	1.85	0.00	0.00	0.00	1.72	0.00	0.00	0.00	0.95
Dicalcium phosphate	0.75	1.00	1.40	1.40	0.60	0.90	1.25	1.30	0.55	0.75	1.20	1.40
Limestone	1.30	1.15	0.95	0.95	1.25	1.10	0.95	0.90	1.20	1.00	0.80	0.70
Salt	0.35	0.35	0.35	0.35	0.35	0.35	0.35	0.35	0.35	0.35	0.35	0.35
L-Lysine•HCl	0.13	0.14	0.15	0.18	0.05	0.07	0.10	0.13	0.07	0.09	0.12	0.13
Vitamin and mineral premix^1^	1.00	1.00	1.00	1.00	1.00	1.00	1.00	1.00	1.00	1.00	1.00	1.00
Calculated composition												
ME, Mcal/kg	3.11	3.20	3.31	3.40	3.11	3.20	3.30	3.40	3.05	3.15	3.25	3.35
SID Lys, %	0.81	0.83	0.87	0.89	0.71	0.74	0.76	0.78	0.61	0.64	0.65	0.67
SID-Lys/ME, g/Mcal	2.60	2.60	2.60	2.60	2.30	2.30	2.30	2.30	2.00	2.00	2.00	2.00
Analyzed composition, %												
Lysine	0.92	0.94	0.97	0.99	0.83	0.84	0.86	0.88	0.72	0.73	0.74	0.76
Methionine	0.24	0.25	0.25	0.26	0.23	0.24	0.25	0.24	0.20	0.22	0.21	0.22
Threonine	0.66	0.67	0.70	0.70	0.64	0.66	0.65	0.65	0.55	0.56	0.55	0.57
Tryptophan	0.21	0.21	0.22	0.22	0.20	0.19	0.19	0.19	0.17	0.17	0.17	0.16
Calcium	0.73	0.75	0.75	0.74	0.70	0.71	0.69	0.71	0.64	0.63	0.65	0.62
Phosphorus	0.64	0.62	0.63	0.63	0.60	0.61	0.60	0.59	0.60	0.58	0.59	0.58

^1 ^Vitamin and mineral premix provided the following per kg of feed:

30 to 50 kg: Vitamin A, 6,000 IU; Vitamin D_3_, 2,400 IU; Vitamin E, 21.6 IU; Vitamin K_3_, 2 mg; Vitamin B_1_, 0.96 mg; Vitamin B_2_, 5.2 mg; Vitamin B_6_, 2 mg; Vitamin B_12_, 12 μg; Nicotinic acid, 22 mg; Pantothenic acid, 11.2 mg; Folic acid, 0.4 mg; Biotin, 40 μg; Choline chloride, 0.4 g; Fe, 120 mg; Cu, 140 mg; Zn, 100 mg; Mn, 16 mg; I, 0.24 mg; Se, 0.4 mg.

52 to 70 kg: Vitamin A, 5,600 IU; Vitamin D_3_, 2,200 IU; Vitamin E, 21.6 IU; Vitamin K_3_, 1.8 mg; Vitamin B_1_, 0.88 mg; Vitamin B_2_, 4 mg; Vitamin B_6_, 1.8 mg; Vitamin B_12_, 12 μg; Nicotinic acid, 20 mg; Pantothenic acid, 10 mg; Folic acid, 0.4 mg; Biotin, 40 μg; Choline chloride, 0.32 g; Fe, 88 mg; Cu, 120 mg; Zn, 96 mg; Mn, 16 mg; I, 0.24 mg; Se, 0.4 mg.

81 to 104 kg: Vitamin A, 5,200 IU; Vitamin D_3_, 2,000 IU; Vitamin E, 17.2 IU; Vitamin K_3_, 1.6 mg; Vitamin B_1_, 0.8 mg; Vitamin B_2_, 3.6 mg; Vitamin B_6_, 1.6 mg; Vitamin B_12_, 10 μg; Nicotinic acid, 17.6 mg; Pantothenic acid, 8.8 mg; Folic acid, 0.38 mg; Biotin, 32 μg; Choline chloride, 0.24 g; Fe, 76 mg; Cu, 120 mg; Zn, 76 mg; Mn, 12 mg; I, 0.24 mg; Se, 0.4 mg.

For Exps 4 to 6, a total of 450, 375 and 360 crossbred pigs (Duroc × Landrace × Large White), weighing 28.98 ± 5.41 kg, 53.95 ± 6.06 kg and 85.45 ± 8.14 kg of BW were assigned to one of five treatments. The treatments consisted of diets with a SID-Lys:ME ratio of 2.4, 2.6, 2.8, 3.0 or 3.2 for Exp. 1, 2.1, 2.3, 2.5, 2.7, 2.9 or 3.2 for Exp. 2 and 1.8, 2.0, 2.2, 2.4, or 2.6 for Exp. 3. A constant ME level 3.2, 3.2 and 3.05 Mcal/kg was used in Exps. 1, 2 and 3, respectively (Table 3). Other amino acids were balanced relative to lysine using crystalline amino acids in order to match the ideal amino acid profile of the Feeding Standard of Swine [2].

**Table 3:** Ingredient and chemical composition of experimental diets used in experiments 4 to 6 (as-fed)

Item	Lys/ME in 29 to 47 kg, g/Mcal					Lys/ME in 54 to 76 kg, g/Mcal					Lys/ME in 84 to 109 kg, g/Mcal				
	
	2.40	2.60	2.80	3.00	3.20	2.10	2.30	2.50	2.70	2.90	1.80	2.00	2.20	2.40	2.60
Ingredient, %															
Corn	63.00	63.00	63.00	63.00	63.00	65.35	65.00	65.00	65.00	65.00	60.50	60.28	60.28	60.28	60.28
Soybean meal (43% CP)	24.00	24.00	24.00	24.00	24.00	21.80	22.08	22.08	22.08	22.08	15.00	15.50	15.50	15.50	15.50
Wheat bran	9.44	9.36	9.21	9.04	8.89	9.50	9.50	9.39	9.22	9.06	21.40	21.05	20.94	20.81	20.64
Soybean oil	0.00	0.00	0.00	0.00	0.00	0.00	0.00	0.00	0.00	0.00	0.00	0.00	0.00	0.00	0.00
Dicalcium phosphate	1.00	1.00	1.00	1.00	1.00	0.90	0.90	0.90	0.90	0.90	0.55	0.55	0.55	0.55	0.55
Limestone	1.15	1.15	1.15	1.15	1.15	1.10	1.10	1.10	1.10	1.10	1.20	1.20	1.20	1.20	1.20
Salt	0.35	0.35	0.35	0.35	0.35	0.35	0.35	0.35	0.35	0.35	0.35	0.35	0.35	0.35	0.35
L-Lysine•HCl	0.06	0.14	0.22	0.30	0.38	0.00	0.07	0.15	0.23	0.32	0.00	0.07	0.15	0.22	0.30
MHA^1^	0.00	0.00	0.02	0.04	0.06	0.00	0.00	0.01	0.04	0.06	0.00	0.00	0.01	0.02	0.05
L-Threonine	0.00	0.00	0.04	0.09	0.13	0.00	0.00	0.02	0.06	0.10	0.00	0.00	0.02	0.06	0.11
L-Tryptophan	0.00	0.00	0.01	0.03	0.04	0.00	0.00	0.00	0.02	0.03	0.00	0.00	0.00	0.01	0.02
Vitamin and mineral premix^2^	1.00	1.00	1.00	1.00	1.00	1.00	1.00	1.00	1.00	1.00	1.00	1.00	1.00	1.00	1.00
Calculated composition															
SID Lys (%)	0.77	0.83	0.88	0.96	1.02	0.68	0.74	0.80	0.86	0.93	0.55	0.61	0.68	0.73	0.79
SID Lys/ME (g/Mcal)	2.4	2.6	2.8	3.0	3.2	2.1	2.3	2.5	2.7	2.9	1.8	2.0	2.2	2.4	2.6
ME (Mcal/kg)	3.20	3.20	3.20	3.20	3.20	3.20	3.20	3.20	3.20	3.19	3.05	3.06	3.06	3.05	3.05
Analyzed composition (%)															
Lysine	0.89	0.94	1.00	1.07	1.14	0.79	0.84	0.89	0.97	1.04	0.65	0.73	0.80	0.84	0.90
Methionine	0.25	0.25	0.27	0.28	0.31	0.24	0.26	0.26	0.28	0.29	0.20	0.21	0.23	0.23	0.24
Threonine	0.68	0.69	0.72	0.77	0.81	0.65	0.65	0.66	0.71	0.74	0.55	0.55	0.56	0.60	0.66
Tryptophan	0.21	0.21	0.22	0.24	0.24	0.21	0.20	0.21	0.22	0.23	0.17	0.18	0.17	0.18	0.19
Calcium	0.75	0.75	0.75	0.74	0.74	0.71	0.70	0.70	0.71	0.70	0.63	0.65	0.65	0.63	0.63
Phosphorus	0.63	0.62	0.63	0.64	0.63	0.61	0.61	0.60	0.62	0.60	0.61	0.60	0.59	0.62	0.61

^1 ^DL-methionine hydroxy analogue (84%), brand name of Novus International Inc., St. Louis, MO.

^2 ^Vitamin and mineral premix provided the following per kg of feed:

29 to 47 kg: Vitamin A, 6,000 IU; Vitamin D_3_, 2,400 IU; Vitamin E, 21.6 IU; Vitamin K_3_, 2 mg; Vitamin B_1_, 0.96 mg; Vitamin B_2_, 5.2 mg; Vitamin B_6_, 2 mg; Vitamin B_12_, 12 μg; Nicotinic acid, 22 mg; Pantothenic acid, 11.2 mg; Folic acid, 0.4 mg; Biotin, 40 μg; Choline chloride, 0.4 g; Fe, 120 mg; Cu, 140 mg; Zn, 100 mg; Mn, 16 mg; I, 0.24 mg; Se, 0.4 mg.

54 to 76 kg: Vitamin A, 5,600 IU; Vitamin D_3_, 2,200 IU; Vitamin E, 21.6 IU; Vitamin K_3_, 1.8 mg; Vitamin B_1_, 0.88 mg; Vitamin B_2_, 4 mg; Vitamin B_6_, 1.8 mg; Vitamin B_12_, 12 μg; Nicotinic acid, 20 mg; Pantothenic acid, 10 mg; Folic acid, 0.4 mg; Biotin, 40 μg; Choline chloride, 0.32 g; Fe, 88 mg; Cu, 120 mg; Zn, 96 mg; Mn, 16 mg; I, 0.24 mg; Se, 0.4 mg.

84 to 109 kg: Vitamin A, 5,200 IU; Vitamin D_3_, 2,000 IU; Vitamin E, 17.2 IU; Vitamin K_3_, 1.6 mg; Vitamin B_1_, 0.8 mg; Vitamin B_2_, 3.6 mg; Vitamin B_6_, 1.6 mg; Vitamin B_12_, 10 μg; Nicotinic acid, 17.6 mg; Pantothenic acid, 8.8 mg; Folic acid, 0.38 mg; Biotin, 32 μg; Choline chloride, 0.24 g; Fe, 76 mg; Cu, 120 mg; Zn, 76 mg; Mn, 12 mg; I, 0.24 mg; Se, 0.4 mg.

Each experiment used a randomized complete block design experiment which was conducted for 28 days. Each treatment was applied to six pens with 11 to 15 pigs (half male, half female) per pen. Each pen (3 × 4.5 m^2^) was equipped with a nipple waterer and two-hole dry feeder. Individual pigs and feeders were weighed at the beginning and the end of the experiment and these values were used to calculate weight gain, feed intake and feed efficiency.

At the end of Exps. 3 and 6, one pig was selected randomly from each pen to be slaughtered to determine carcass characteristics (left side of each carcass). Hot carcass weight (dressing percentage = carcass/body weight), tenth rib back fat thickness, and loin eye (0.7 × loin eye width × depth, cm^2^) were measured. The pH of the carcass was measured with a pH meter (HI 8424NEW, HANNA, Rome, Italy) 45 min after slaughter and again 24 h after being placed in a 4°C refrigerator. Meat color was determined with a Chromameter, CR 400, (Minolta; Tokyo, Japan).

### Chemical analysis

At the beginning of each experiment, feed samples were collected and ground to pass through a 1.0-mm screen (40 mesh). Analyses for dry matter, calcium, and total phosphorus were conducted according to the methods of AOAC [13]. Gross energy was measured by an automatic adiabatic oxygen bomb calorimeter (Parr 6300 Calorimeter; Moline, IL, USA).

The amino acid concentration in diets was analyzed after the diets were ground through a 60 mesh screen. Feed samples were hydrolyzed in 6 N HCl (10 mL) at 110°C for 24 h under nitrogen. Sulphur containing amino acids were measured after performic acid oxidation [13]. Trytophan content was determined colorimetrically after alkaline hydrolysis following the procedures described by Miller [14]. Then the amino acids were analyzed by using a S-433D Amino Acid Analyzer (Sykam GmbH; Kleinostheim, Germany). Identification and quantification of amino acids were achieved by comparing the retention times of the peaks with those of standards.

### Statistical analysis

The pen was the experimental unit for all analyses. Data were analyzed by ANOVA using a randomized complete block design according to the GLM procedure of SAS (SAS Inst. Inc.; Cary, NC, USA). Blocks were based on initial body weight. Total variation was divided into treatment, block and error as follows:

$$\begin{array}{c} {\text{Y}}_{\text{ij}}=\mu +{\text{T}}_{\text{ij}}+{\text{R}}_{\text{ij}}+\xi_{\text{ij}}\\ \text{(i}=\text{1},\text{ 2}\;...,\text{ a},\text{ j}=\text{1},\text{ 2}\;...,\text{ b)} \end{array}$$

Where Y_ij _is the observed value, μ is the population mean, T_ij _is the treatment effect, R_ij _is block effect, ξ_ij _is the random error, a is the number of treatments (4 or 5), b is the number of blocks (6 in each experiment). Statistical differences among treatments were separated by Duncan's Multiple Range Test. Statistical significance was declared at *P *< 0.05 and a trend was expressed when *P *< 0.10. Results are presented as least squares means. In Exps. 4, 5 and 6, a straight broken line regression model with a random component included for parameter L (LVAR, represents the block effect and produces different lines for the respective blocks) was used to estimate a break-point according to Robbins et al. [15] using the NLMixed procedure of SAS [16].

## Results and discussion

The effects of increasing ME level on the performance of pigs during the growing, early finishing and late finishing stages are shown in Table 4. Weight gain was significantly increased (*P *< 0.05) with increasing energy level in Exp.1 (30 to 50 kg) while weight gain was unaltered in Exps. 2 (52 to 70 kg) and 3 (81 to 104 kg). But weight gain tended (*P *< 0.10) to greater in ME level of 3.2 and 3.3 than 3.1 Mcal/kg in Exp. 2. Liu et al. [17] reported increased weight gains when the dietary energy increased which is similar to the results of Du et al. [18]. Similar to the current study, De La Llata et al. [9] found pigs fed high energy levels had a significantly improved weight gain only during the growing stage and not during the finishing stage. Similarly, Beaulieu et al. [4] found that increasing the energy content of diets increased weight gain from 36 to 80 kg but pigs of heavier weight did not respond to increasing energy levels. Kerr et al. [19] observed increased weight gain when dietary net energy levels were increased from 25 to 41 kg, but not in the finishing phase (58 to 82 kg).

**Table 4:** Effect of dietary ME level on performance of growing-finishing pigs (Experiments 1, 2 and 3)^1^

Item	ME level, Mcal/kg				SEM	*P *value		
						
						ANOVA	Linear	Quadratic
Experiment 1 (30 to 50 kg)	3.1	3.2	3.3	3.4				
					
Weight gain, kg/d	0.64^a^	0.68^b^	0.67^b^	0.67^b^	0.01	0.02	0.09	0.19
Feed intake, kg/d	2.10^a^	1.92^ab^	1.79^b^	1.70^b^	0.07	0.01	0.43	0.01
Feed efficiency	0.31^a^	0.36^b^	0.38^b^	0.40^b^	0.02	< 0.01	0.95	< 0.01
Experiment 2 (52 to 70 kg)	3.1	3.2	3.3	3.4				
					
Weight gain, kg/d	0.74	0.79	0.79	0.76	0.02	0.08	0.57	0.31
Feed intake, kg/d	2.75^a^	2.57^ab^	2.51^ab^	2.33^b^	0.09	0.04	0.06	0.18
Feed efficiency	0.27^a^	0.31^ab^	0.32^b^	0.33^b^	0.01	0.04	0.01	0.03
Experiment 3 (81 to 104 kg)	3.05	3.15	3.25	3.35				
					
Weight gain, kg/d	0.78	0.78	0.78	0.80	0.01	0.53	0.20	0.39
Feed intake, kg/d	3.38^a^	3.27^a^	3.20^a^	2.97^b^	0.06	< 0.01	0.02	0.06
Feed efficiency	0.23^a^	0.24^a^	0.25^a^	0.27^b^	0.01	< 0.01	< 0.01	0.03

^a, b^Means in the same row with different superscripts are significantly different (*P *< 0.05).

^1^Value represent means of six pens with 11 to 15 pigs per pen during a 28-d period.

For all three experiments, feed intake was decreased (*P *< 0.05) and feed efficiency was improved (*P *< 0.05) with increasing energy level. This is consistent with previous reports [4,9,20] which supports the findings of the current experiment. It is well known that pigs will compensate for increases in the energy density of their diet by decreasing their feed intake [21,22].

The effects of increasing ME level on the carcass characteristics of finishing pigs are shown in Table 5. Tenth rib back fat thickness increased linearly (*P *< 0.05) with increasing dietary ME content. Loin area showed a quadratic trend (*P *= 0.06). Loin eye area increased as the dietary ME level was increased to 3.15 to 3.25 Mcal/kg but then declined as the energy level was further increased to 3.35 Mcal/kg. Increasing the dietary ME had no effect on dressing percentage, pH_45 min_, pH_24 h _and meat color.

**Table 5:** Effect of dietary ME level on carcass characteristics of finishing pigs (Experiment 3)^1^

Item	ME, Mcal/kg				SEM	*P *value		
			
	3.05	3.15	3.25	3.35		ANOVA	Linear	Quadratic
Dressing percentage, %	71.98	72.31	73.30	74.03	1.86	0.86	0.34	0.64
10^th^-rib back fat thickness, mm	18.5	21.5	22.3	24.0	1.42	0.09	0.02	0.07
Loin area, cm^2^	39.62	44.42	42.98	40.31	1.59	0.16	0.93	0.06
pH^45 min^	6.42	6.19	6.30	6.30	0.09	0.37	0.48	0.39
pH^24 h^	5.58	5.53	5.50	5.49	0.05	0.58	0.17	0.36
L* (Lightness)	41.45	40.81	40.77	42.58	1.77	0.88	0.67	0.72
a* (Redness)	14.31	13.49	15.61	14.57	0.66	0.20	0.35	0.64
b* (Yellowness)	5.24	4.74	5.54	5.67	0.56	0.65	0.38	0.58

^1^Value represent means of six pens with one pig per pen.

In the literature, the effects of dietary energy on back fat thickness have been shown to be variable. In one experiment of the study of Beaulieu et al. [4], increasing energy did not influence back fat thickness, but in another experiment, back fat thickness increased and loin area declined as dietary energy increased, which is similar to the present study. Apple et al. [23] and Lawrence et al. [24] also observed back fat thickness increased with increasing dietary energy.

The effects of increasing SID-Lys:ME ratio on the performance of pigs during the growing, early finishing and late finishing stages are shown in Table 6. For all three experiments, weight gain increased (*P *< 0.05) and feed efficiency improved linearly (*P *< 0.05) as the SID-Lys:ME ratio increased. In the study of Smith et al. [3], increasing the lysine:ME ratio increased weight gain but did not influence feed efficiency. However, Friesen et al. [25] found weight gain increased and feed efficiency improved while feed intake tended to increase in growing pigs (34 to 72 kg).

**Table 6:** Effect of dietary increasing SID-Lys:ME ratio on performance of growing-finishing pigs (Experiments 4, 5 and 6)^1^

Item	SID-Lys:ME ratio, g/Mcal					SEM	*P *value		
						
							ANOVA	Linear	Quadratic
Experiment 4 (29 to 47 kg)	2.4	2.6	2.8	3.0	3.2				
					
Weight gain, kg/d	0.62^a^	0.64^ab^	0.64^ab^	0.68^b^	0.66^b^	0.01	0.04	0.09	0.22
Feed intake, kg/d	1.85	1.80	1.76	1.74	1.73	0.06	0.66	0.34	0.61
Feed efficiency	0.34	0.36	0.37	0.39	0.38	0.02	0.24	0.04	0.09
Experiment 5 (54 to 76 kg)	2.1	2.3	2.5	2.7	2.9				
					
Weight gain, kg/d	0.73^a^	0.77^b^	0.80^b^	0.80^b^	0.79^b^	0.01	< 0.01	0.01	0.01
Feed intake, g/d	2.44	2.44	2.38	2.35	2.34	0.08	0.86	0.49	0.79
Feed efficiency	0.30	0.32	0.34	0.34	0.34	0.01	0.16	0.05	0.10
Experiment 6 (84 to 109 kg)	1.8	2.0	2.2	2.4	2.6				
					
Weight gain, kg/d	0.79^ab^	0.76^a^	0.88^c^	0.86^c^	0.84^bc^	0.03	< 0.01	0.03	0.06
Feed intake, kg/d	3.26	3.20	3.34	3.35	3.15	0.10	0.24	0.86	0.57
Feed efficiency	0.24	0.24	0.26	0.26	0.27	0.01	0.07	0.02	0.08

^a-c^Means in the same row with different superscripts are significantly different (*P *< 0.05).

^1^Value represent means of six pens with 11 to 15 pigs per pen during a 28-d period.

Chiba et al. [26] and Rao and McCracken [27] both found increased weight gain and feed efficiency in growing pigs when the lysine:energy ratio increased. [1] observed increased weight gain and improved feed efficiency with increasing dietary Lys:ME ratio both in growing-finishing barrows and gilts. This is consistent with present study.

The effects of increasing SID-Lys:ME ratio on carcass characteristics of finishing pigs are shown in Table 7. Tenth rib back fat thickness was decreased (linear, *P *< 0.05; Quadratic, *P *< 0.05) as the SID-Lys:ME ratio increased. Loin area increased (*P *< 0.05) as the SID-Lys:ME ratio increased. Increasing the dietary SID-Lys/ME ratio had no effect on dressing percentage, pH_45 min_, pH_24 h _and meat colors. Smith et al. [3] reported that carcass characteristics were not influenced by dietary lysine to energy ratio. However, in other studies, increasing the lysine to energy ratio decreased body fat and increased loin area [1,28,29] which somewhat supports the findings of the current experiment.

**Table 7:** Effect of dietary increasing SID-Lys:ME ratio on carcass characteristics of finishing pigs (Experiment 6)

	SID-Lys:ME ratio, g/Mcal					SEM	*P *value		
			
	1.8	2.0	2.2	2.4	2.6		ANOVA	Linear	Quadratic
Dressing percentage, %	73.04	73.25	74.25	74.99	75.33	1.62	0.81	0.18	0.42
10^th^-rib back fat thickness, mm	21.00^a^	21.67^a^	20.00^a^	19.67^a^	17.17^b^	0.82	0.01	0.02	0.03
Loin area, cm^2^	40.55^a^	40.87^a^	41.84^ab^	41.29^a^	43.34^b^	0.63	0.04	0.09	0.22
pH^45 min^	6.63	6.31	6.15	6.64	6.65	0.26	0.53	0.65	0.35
pH^24 h^	5.35	5.54	5.45	5.39	5.53	0.22	0.96	0.72	0.94
L* (Lightness)	42.81	43.09	42.01	44.5	43.05	1.71	0.89	0.73	0.94
a* (Redness)	13.48	12.97	14.7	13.35	13.89	0.75	0.56	0.58	0.80
b* (Yellowness)	5.77	5.26	6.04	6.23	6.11	0.52	0.70	0.29	0.58

^a, b^Means in the same row with different superscripts are significantly different (*P *< 0.05).

^1^Value represent means of six pens with one pig per pen.

**Figure 1:**
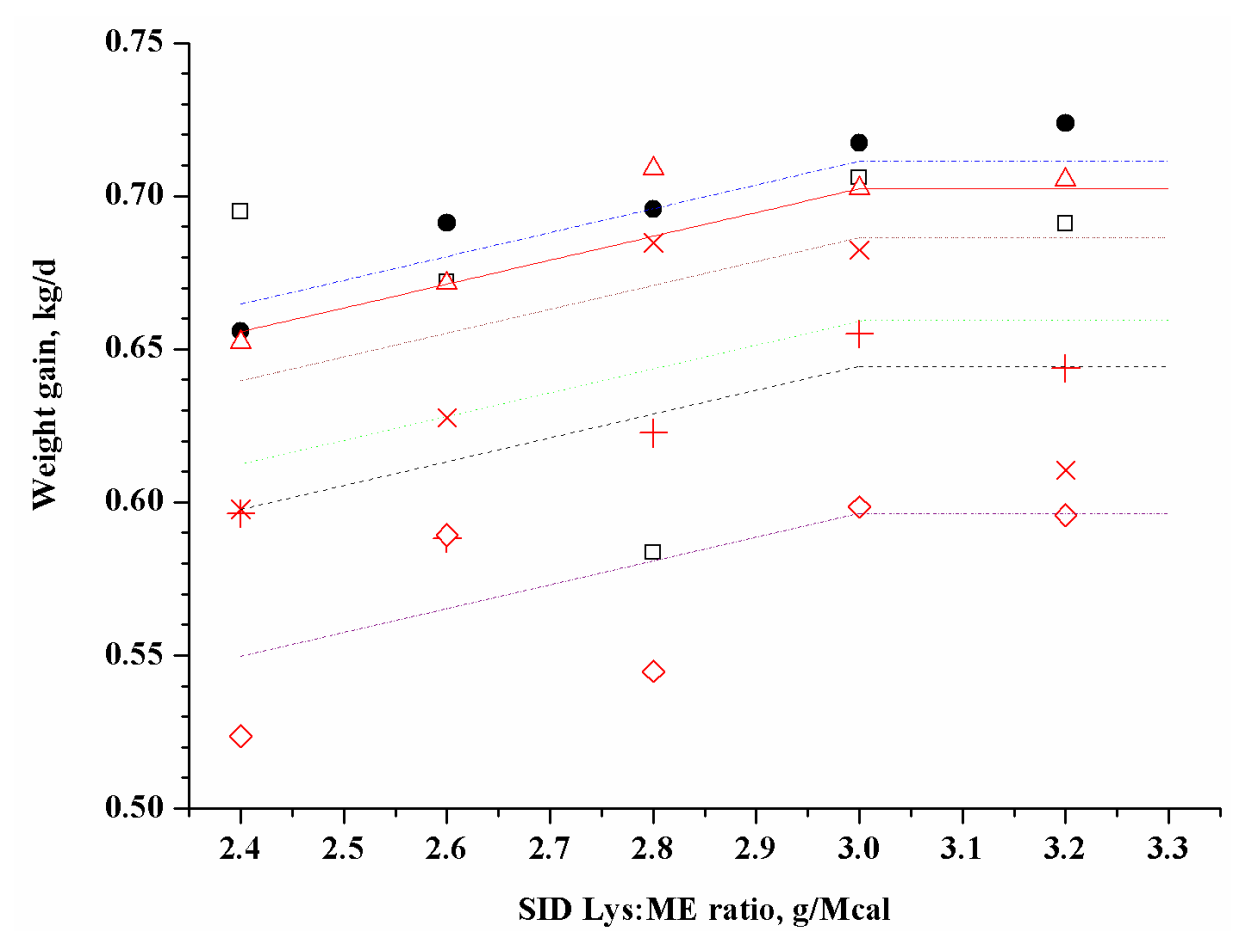
**Fitted straight broken-line of weight gain as a function of SID-Lys:ME ratio (Exp. 4)**. Observed block mean values in each treatment (dot represent block 1, square represent block 2, triangle represent block 3, × represent block 4, plus represent block 5, DIAMOND represent block 6) are shown. The straight broken line: y = 0.6664 - 0.07787 × (3 - x) + LVAR (r^2^ = 0.76, adjusted r^2^ = 0.64). Parameter LVAR represents the block effect and produces different lines for the respective blocks. The optimum SID-Lys:ME ratio was 3 in 28 to 47 kg pig.

**Figure 2:**
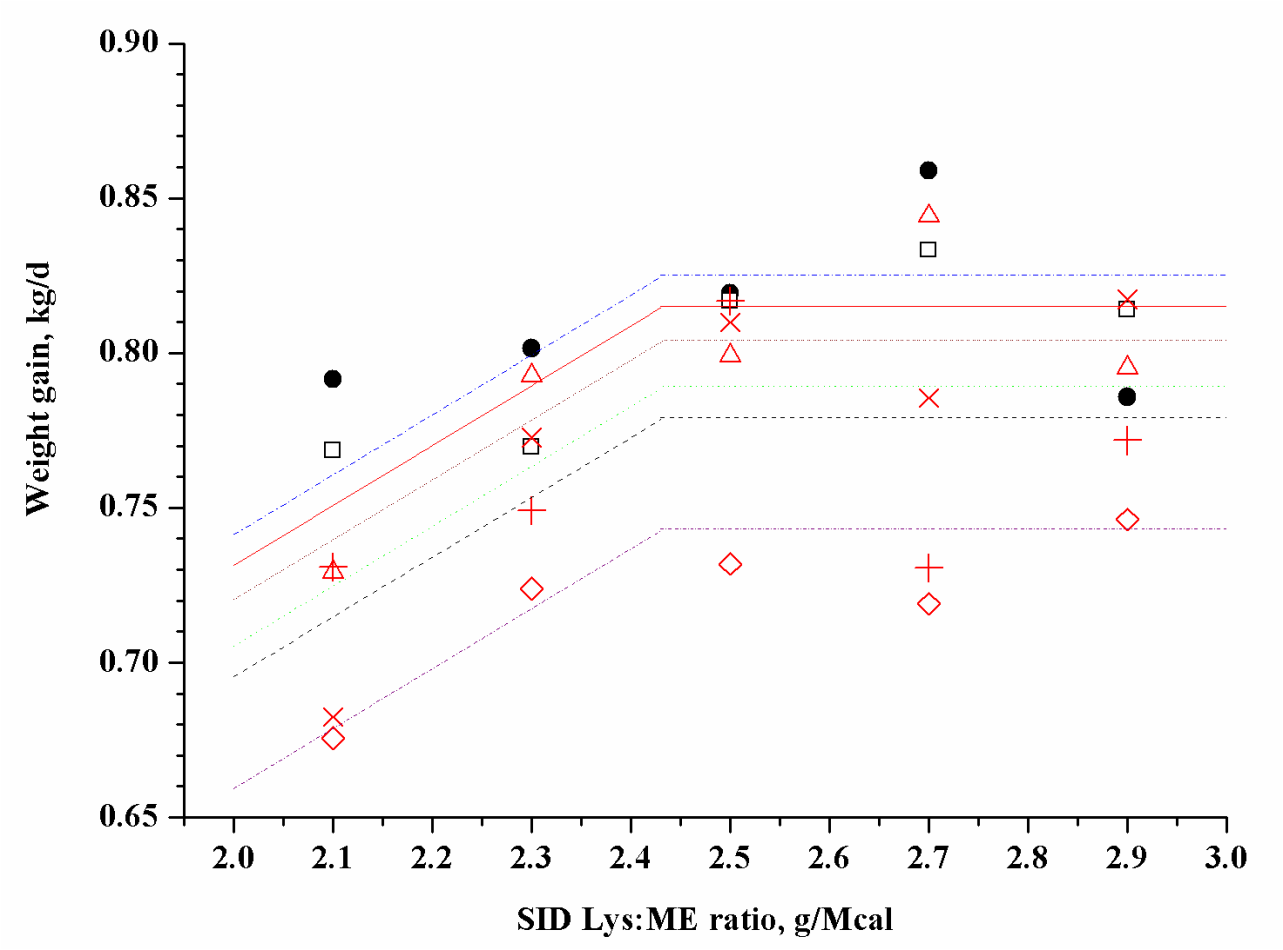
**Fitted straight broken-line of weight gain as a function of SID-Lys:ME ratio (Exp. 5)**. Observed block mean values in each treatment (dot represent block 1, square represent block 2, triangle represent block 3, × represent block 4, plus represent block 5, DIAMOND represent block 6) are shown. The straight broken line: y = 0.7942 - 0.1933 × (2.4336 - x) + LVAR (r^2^ = 0.76, adjusted r^2^ = 0.63). Parameter LVAR represents the block effect and produces different lines for the respective blocks. The optimum SID-Lys:ME ratio was 2.43 in 54 to 76 kg pig.

**Figure 3:**
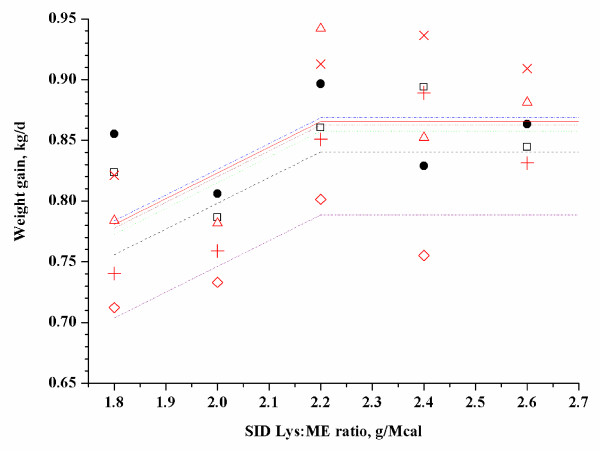
**Fitted straight broken-line of weight gain as a function of SID-Lys:ME ratio (Exp. 6)**. Observed block mean values in each treatment (dot represent block 1, square represent block 2, triangle represent block 3, × represent block 4, plus represent block 5, DIAMOND represent block 6) are shown. Two observations (× in treatment two and DIAMOND in treatment five) were considered outliers because they were influential points in the model. The straight broken line: y = 0.8587 - 0.209 × (2.2 - x) + LVAR (r^2^ = 0.74, adjusted r^2^ = 0.59). Parameter LVAR represents the block effect and produces different lines for the respective blocks. The optimum SID Lys:ME ratio was 2.2 in 84 to 109 kg pig.

The data from Exps. 4 to 6 were fitted to a straight broken-line regression equation:

$$\text{y}=0.6664-0.07787\times \left(\text{3}.0-\text{x}\right)+\text{LVAR}$$

(r^2 ^= 0.76, adjusted r^2 ^= 0.64; Exp. 4, Figure 1)

$$\text{y}=0.\text{7942}-0.\text{1933}\times \left(\text{2}.\text{43}-\text{x}\right)+\text{LVAR}$$

(r^2 ^= 0.76, adjusted r^2 ^= 0.63; Exp. 5, Figure 2)

$$\text{y}=0.\text{8587}-0.\text{2}0\text{9}\times \left(\text{2}.\text{2 }-\text{ x}\right)+\text{LVAR}$$

(r^2 ^= 0.74, adjusted r^2 ^= 0.59; Exp. 6, Figure 3)

Parameter LVAR represents the block effect and produced different lines for the respective blocks. Based on a straight broken-line model, the estimated SID-Lys:ME ratio to optimize weight gain was 3.0, 2.43 and 2.2 for 29 to 47 kg, 54 to 76 kg and 84 to 109 kg of pigs, respectively. In addition, the estimated total Lys:ME ratio to optimize weight gain was 3.34, 2.69 and 2.61 for 29 to 47 kg, 54 to 76 kg and 84 to 109 kg of pigs, respectively.

Main et al. [1] estimated 3.14, 2.66 and 2.2 g/Mcal of total Lys:ME ratio for 43 to 70 kg, 69 to 93 kg and 102 to 120 kg barrows, respectively; and 3.23, 2.8, 2.28 and 2.2 g/Mcal of total Lys:ME ratio for 35 to 60 kg, 60 to 85 kg, 78 to 103 kg and 100 to 120 kg gilts, respectively. Yen et al. [30] estimated 3.13 and 3.23 g/Mcal of total Lys:ME ratio for 20 to 55 kg barrows and gilts respectively. Chang et al. [6] reported that the optimum total Lys:ME ratio for 16 to 57 kg barrows was 3.3 g/Mcal. Yen et al. [31] estimated 2.34 and 2.73 g/Mcal of total Lys:ME ratio for 50 to 90 kg barrows and gilts respectively. Friesen et al. [25] observed 2.78 g/Mcal of total Lys:ME ratio for 55 to 72 kg gilts. Hahn et al. [32] reported that the optimum total Lys:ME ratio for 90 to 110 kg gilts was 1.77 g/Mcal.

In conclusion, based on our results, we suggest the optimum ratio of SID-Lys:ME is 3.0, 2.43 and 2.2 g/Mcal for 29 to 47 kg, 54 to 76 kg and 84 to 109 kg of pigs, respectively.

## Competing interests

The authors declare that they have no competing interests.

## Authors' contributions

PL carried out the experiments and drafted the manuscript. ZZ performed the statistical analysis. DW, LX and RZ participated in the design of the study. XP conceived of the study, and participated in its design and coordination. All authors read and approved the final manuscript.
